# Das Netzwerk Universitätsmedizin: Technisch-organisatorische Ansätze für Forschungsdatenplattformen

**DOI:** 10.1007/s00103-022-03649-1

**Published:** 2023-01-23

**Authors:** Ralf Heyder, NUM Coordination Office, Heyo K. Kroemer, Silke Wiedmann, Christina Pley, Carolin Heyer, Peter Heuschmann, Jörg Janne Vehreschild, Dagmar Krefting, Thomas Illig, Matthias Nauck, Jens Schaller, Monika Kraus, Wolfgang Hoffmann, Dana Stahl, Sabine Hanß, Gabriele Anton, Christian Schäfer, Jens-Peter Reese, Sina M. Hopff, Roberto Lorbeer, Bettina Lorenz-Depiereux, Hans-Ulrich Prokosch, Sven Zenker, Roland Eils, Andreas Bucher, Jens Kleesiek, Thomas Vogl, Bernd Hamm, Tobias Penzkofer, Wiebke Schirrmeister, Rainer Röhrig, Felix Walcher, Raphael Majeed, Bernadett Erdmann, Simone Scheithauer, Hajo Grundmann, Alexander Dilthey, Anna Bludau, NUKLEUS Study Group, NUM-RDP Coordination, RACOON Coordination, AKTIN Coordination, GenSurv Study Group

**Affiliations:** NUM Coordination Office, Charité – Universitätsmedizin Berlin, Corporate member of Freie Universität Berlin and Humboldt-Universität zu Berlin, Charitéplatz, 10117 Berlin, Deutschland

**Keywords:** NUM, Pandemic Preparedness, Forschungsinfrastruktur, COVID-19, FAIR Data Principles, Datenbank, Pandemic Preparedness, Research Infrastructure, COVID-19, FAIR Data Principles, Database

## Abstract

Das Netzwerk Universitätsmedizin (NUM) besteht aus den 36 Standorten der Universitätsmedizin in Deutschland. Der Auftrag ist die Koordinierung der universitätsmedizinischen COVID-19-Forschung auf nationaler Ebene. Dazu werden u. a. gemeinsame Infrastrukturen für die Sammlung, Haltung und Nutzung medizinischer Forschungsdaten benötigt. Diese standen beim Start des NUM-Projekts im April 2020 nicht im erforderlichen Rahmen zur Verfügung. Medizinische Forschungsdaten sind extrem heterogen und gehen weit über „Real World Data“ (Daten aus dem Versorgungsalltag) hinaus. Eine universelle Lösung dafür gab es nicht, deshalb hat das NUM fünf Forschungsinfrastrukturen für unterschiedliche Datenarten, unterschiedliche Wege der Datengewinnung und unterschiedliche Datenentstehungssettings aufgebaut. Um die Bildung neuer Datensilos zu verhindern, arbeiten alle fünf Infrastrukturen auf Basis der FAIR-Prinzipien, nach denen Daten auffindbar (findable), zugänglich (accessible), interoperabel (interoperable) und wiederverwendbar (reusable) sein sollen. Zudem implementiert das NUM einen übergreifenden Steuerungsrahmen (Governance Framework), um die Weiterentwicklung dieser fünf Infrastrukturen zentral zu steuern. Der Artikel beschreibt den aktuellen Stand der Infrastrukturentwicklung im NUM und mögliche Perspektiven. Ein starker Fokus wird dabei auf die technisch-organisatorischen Grundlagen gerichtet.

## Abstract (en)

The Network University Medicine (NUM) consists of 36 university clinics in Germany. It was set up to coordinate COVID-19 university medicine research activities on a national level. This required, among other things, common infrastructures for the collection, storage, and use of medical research data. These infrastructures were not available in the required form when the NUM started in April 2020. Medical research data are extremely heterogeneous and reach far beyond “real world data” from patient care. There was no “one size fits all” solution, so NUM built five infrastructures for different types of data, different ways of obtaining data, and different data origination settings. To prevent the creation of new data silos, all five infrastructures operate based on FAIR principles (findable, accessible, interoperable, reusable). In addition, NUM is implementing an overarching governance framework to manage the evolution of these five infrastructures. The article describes the current state of development and possible perspectives with a strong focus on technical and organizational aspects.

## Einleitung

Das Netzwerk Universitätsmedizin (NUM) wurde im April 2020 im Rahmen einer Projektförderung des Bundesministeriums für Bildung und Forschung (BMBF) als Reaktion auf die erste COVID-19-Infektionswelle gegründet. Aufgabe ist die Koordination der COVID-19-Forschung der Universitätsmedizin mit dem Ziel, Patient*innenversorgung und Pandemiemanagement möglichst unmittelbar mit Evidenz zu unterstützen und dabei gut abgestimmt zu agieren. Das NUM setzt somit am Ende der Translationskette an. Auf Bundesebene ist das NUM die erste Initiative zur interdisziplinären Vernetzung, Zusammenarbeit und Koordination im Bereich der patientenorientierten Forschung, an der alle deutschen Universitätskliniken (UK) beteiligt sind. Das NUM setzt auf einen partnerschaftlich-kooperativen, nichtkompetitiven Ansatz. Ziel ist u. a. die Stärkung der „pandemic preparedness“ des Versorgungs- und Forschungssystems. Dazu gehört insbesondere der Aufbau geeigneter Forschungsinfrastrukturen (FIS).^1^ Dafür benötigt das NUM drei wesentliche Grundlagen:

- arbeitsfähige standortübergreifende interdisziplinäre Expert*innennetzwerke^2^, die die relevanten Teilaspekte des Pandemiegeschehens organisieren,
- standortübergreifende abgestimmte Sammlung von Forschungsdaten zu COVID-19 und deren Bereitstellung für die wissenschaftliche Gemeinschaft,
- übergreifende, alle UK umfassende Organisationsstrukturen.

Das NUM hat in der 1. Förderperiode (01.04.2020–31.12.2021) in wenigen Wochen 13 große, jeweils als standortübergreifende Verbünde konzipierte Projekte zu unterschiedlichen Aspekten des Pandemiegeschehens auf den Weg gebracht.^3^ Diese Projekte wurden jeweils von unterschiedlichen Expert*innengruppen durchgeführt. Es wurde dabei schnell deutlich, dass in Deutschland zu Beginn der Pandemie die notwendigen Plattformen fehlten, um über die 36 UK hinweg gemeinsam Forschungsdaten strukturiert zu erheben, zu dokumentieren und zu teilen. Deshalb haben einige Projekte FIS entweder neu aufgebaut oder bereits vorhandene FIS gemäß den jeweiligen Anforderungen und Bedürfnissen ausgebaut.

Fünf dieser entstandenen NUM-FIS, die unterschiedliche Arten von medizinischen Forschungsdaten adressieren, werden in der seit 01.01.2022 angelaufenen 2. Förderperiode in der neben den Forschungsprojekten neu etablierten „Infrastrukturlinie“ fortgeführt:

1. *NUM Klinische Epidemiologie- und Studienplattform (NUKLEUS):* Diese Plattform unterstützt die standardisierte Erhebung und Bereitstellung von prospektiv im Rahmen klinischer (Beobachtungs‑)Studien gewonnenen Daten, Bildern, Bioproben und daraus gewonnener Informationen. Sie setzt technisch und konzeptionell auf der Studienplattform des Deutschen Zentrums für Herz-Kreislauf-Forschung e. V. (DZHK) auf, welches in der 1. Förderphase eine schnelle Anpassung dieser Plattform an die Bedürfnisse des NUM unterstützt hat.
2. *NUM-Routinedatenplattform (NUM-RDP):* Sie zielt auf die (retrospektive) Gewinnung von Behandlungsdaten zu COVID-19 aus den klinischen Primärsystemen ab. Eine Ergänzung um Bildgebungs- und Bioprobendaten ist vorgesehen. Das Projekt wird in enger Kooperation mit der Medizininformatik-Initiative (MII) durchgeführt und setzt auf deren Vorarbeiten auf, indem es u. a. die Datenintegrationszentren (DIZ) durch eine zentrale Datenhaltungskomponente ergänzt und verbindet. Die DIZ dienen zudem als Datenquelle für das NUM-Dashboard, welches das Pandemiemanagement mit echtzeitnahen Daten aus der Routineversorgung unterstützt.
3. *Radiological Cooperative Network (RACOON):* Auf dieser Plattform haben sich alle universitären Radiologien Deutschlands zusammengeschlossen, um radiologische Bild- und Befunddaten zu COVID-19 standortübergreifend strukturiert zu erfassen und große Datensätze für die gemeinsame Forschung und das Trainieren von Algorithmen verfügbar zu machen.
4. *AKTIN-Notaufnahmeregister (AKTIN@NUM):* Das bestehende Register des „Aktionsbündnisses zur Verbesserung der Kommunikations- und Informationstechnologie in der Intensiv- und Notfallmedizin“ (AKTIN) wurde durch die NUM-Förderung weiter ausgebaut. Es hat eine dezentrale standardisierte und strukturierte Dokumentation in Notaufnahmen geschaffen und stellt Behandlungsdaten aus diesem spezifischen Setting für Forschungsvorhaben bereit.
5. *NUM Genomic Pathogen Surveillance and Translational Research (GenSurv):* Auf dieser Plattform werden Sequenzierungs- und Metadaten von SARS-CoV-2-Varianten gesammelt, um z. B. die Surveillance bzgl. neu auftretender Virusvarianten mit Hilfe von Verbreitungsanalysen zu unterstützen. GenSurv aggregiert komplexe Omics-Datensätze in geotemporaler Auflösung und interagiert eng mit den Datenbanken des Robert Koch-Instituts (RKI).

Sofern aus den weiteren NUM-Forschungsprojekten zusätzliche (Daten‑)FIS entstehen, besteht die Möglichkeit, die genannten fünf NUM-FIS um weitere Komponenten zu erweitern.

Konzeption und Betrieb von FIS im NUM sind an zwei wesentliche Bedingungen geknüpft:

1. Einhaltung der international etablierten „FAIR Data Principles“^4^, sofern anwendbar,
2. Unterstützung des im NUM einheitlich definierten COVID-19-Kerndatensatzes GECCO^5^, sofern sinnvoll.

Entsprechend diesen Vorgaben stehen die im Rahmen von NUM-Projekten erhobenen Daten und Bioproben der Wissenschaftsgemeinschaft zur Beantwortung von Forschungsfragen über die jeweils im Projekt etablierten Use-and-Access-Verfahren zur Verfügung. Die FIS können grundsätzlich – unter Einhaltung der Nutzungsbedingungen – von allen interessierten Wissenschaftler*innen genutzt werden.

## Die fünf Komponenten der NUM-Forschungsdateninfrastruktur

### NUM Klinische Epidemiologie- und Studienplattform (NUKLEUS)

NUKLEUS unterstützt die Planung, Durchführung und Auswertung von multizentrischen klinischen und klinisch-epidemiologischen Studien durch die Bereitstellung einer Forschungsdateninfrastruktur (6 Komponenten) sowie methodischer Infrastrukturkerne (3 Komponenten). Die Plattform umfasst Methodenexpertise, Prozesse und IT-Infrastruktur zur standardisierten und harmonisierten Planung, Erfassung, Verwaltung und Bereitstellung qualitativ hochwertiger klinischer Daten- und Bioprobensammlungen.

**Figure 1:**
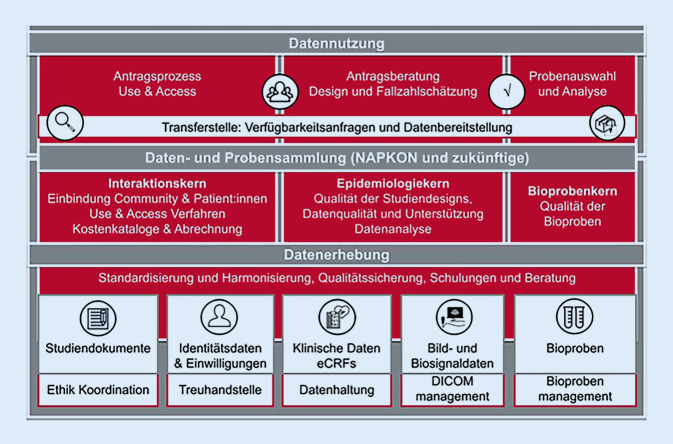

Die Aufgaben von NUKLEUS werden in verschiedenen Arbeitspaketen organisiert (Abb. 1). Dabei übernimmt der *Interaktionskern* (Interaction Core Unit [ICU]) die Kommunikation mit der Forschungsgemeinschaft, den Patient*innenvertretungen und weiteren Stakeholdern und stellt übergreifende Prozesse und Werkzeuge für Nutzer*innen der NUKLEUS-Plattform zur Verfügung; u. a. die Kooperationsplattform, das Use-and-Access-Verfahren und den Kostenkatalog für Studienleistungen inkl. Abrechnung. Der *Epidemiologiekern* (Epidemiology Core Unit [ECU]) sichert die Qualität der in NUKLEUS realisierten Studien durch methodische Beratung zu Planung und Durchführung der Studien und kontinuierlichem Datenqualitätsmonitoring sowie zu Datenanalyse und Datennutzung einschließlich Primärkodierung, sowie Beratungen zum Einsatz von PROMs („patient-reported outcome measures“). Verantwortlich für die Bioprobenqualität und die Standardisierung der assoziierten Daten ist der *Bioprobenkern* (Biosample Core Unit [BCU]), der u. a. Vorgaben zur Probenerhebung und Verwaltung definiert, regelmäßige Audits durchführt sowie die Auswahl herauszugebender Proben und Analysen koordiniert.

Mit dem übergeordneten Ziel der Nachnutzbarkeit steht u. a. die studienübergreifende Standardisierung und Harmonisierung im Vordergrund. Dies umfasst die Erstellung von Studiendokumenten und Ethikanträgen, die von der Ethik-Koordination begleitet werden, sowie die Patient*inneneinwilligungen, die in der Treuhandstelle verwaltet werden. So werden zuverlässig und übergreifend Teilnehmer*innenwille und -rechte gewährleistet. Die erhobenen Daten, Bild‑/Biosignaldaten und Bioproben werden datenschutzkonform in organisatorisch getrennten Systemen pseudonymisiert verwaltet (Prinzip der informationellen Gewaltenteilung). Zur Erfassung der klinischen Daten dient eine kommerzielle Datenerfassungssoftware für klinische Studien (SecuTrial), die Metadaten der Bioproben werden in einem Laborinformationssystem (CentraXX) verwaltet. Die Transferstelle ist für die technische Umsetzung von Verfügbarkeitsanfragen und Datenherausgaben verantwortlich.

Die Infrastrukturkomponenten von NUKLEUS wurden in der 1. Förderphase erfolgreich für die Umsetzung von drei Kohortenstudien zum besseren Verständnis und damit für die Bewältigung von Pandemien am Beispiel der COVID-19-Erkrankung etabliert [1, 2]. Bis Mitte Juli 2022 konnten 5.773 Patient*innen eingeschlossen und diese mit bis zu jeweils 5.200 Datenpunkten charakterisiert werden. Es wurden bereits 89 Datennutzungsanträge bewilligt sowie 36.365 Datensätze und über 42.000 Bioproben herausgegeben. Auf der NUM-Community-Plattform werden momentan 29 fach- und organspezifische Arbeitsgruppen sowie eine AG Patient*innenvertretung koordiniert, die in einem übergreifenden Fachbeirat gemeinsam zum Netzwerk beitragen. Die im Rahmen von NUKLEUS geschaffenen Strukturen zielen darauf ab, dass Studien zu den besten Ideen für die wichtigsten medizinischen Fragestellungen innerhalb weniger Wochen in hoher Qualität realisiert werden können und dadurch Antworten mit hoher Aussagekraft liefern. Das wird ermöglicht durch die einsatzbereite, skalierbare und leistungsfähige Infrastruktur und die Mitwirkung von führenden Expert*innen der Universitätsmedizin.

### NUM-Routinedatenplattform (NUM-RDP)

Die NUM-RDP zielt darauf ab, eine generische Routinedatenplattform mit COVID-19-Bezug (GECCO-Datensatz) bereitzustellen. „Routinedaten“ meint hier Daten der klinischen Routinedokumentation aus der Patient*innenversorgung. In der 1. Förderperiode hat das NUM die in den bereits existierenden MII-Strukturen vorhandene Möglichkeit der föderierten Datenhaltung und -analyse um die Option, Daten zentral einrichtungsübergreifend zusammenzuführen, zu halten und herauszugeben, ergänzt. Diese zentrale Dateninfrastruktur^6^ wird zukünftig um eine Datenmanagementstelle^7^ erweitert. Dies ermöglicht mittelfristig die Durchführung von Verbundforschungsprojekten^8^, indem Daten zu allen Kerndatensatzmodulen von NUM-RDP und MII von den DIZ entgegengenommen und für Broad-Consent-basierte Forschungsprojekte zur Verfügung gestellt werden [3]. Darüber hinaus unterstützt das NUM-Dashboard das Pandemiemanagement mit echtzeitnahem zentralem Tracking der Versorgungsaufwände und Patient*inneneigenschaften.

Die NUM-RDP schafft zwischen den Infrastrukturbetreibern und den UK einen einheitlichen, modular erweiterbaren, generischen, technischen, organisatorischen und rechtlichen Rahmen. Dieser erlaubt eine kurzfristige Anpassung an neue, ad hoc formulierte Anforderungen bspw. für das Pandemiemanagement oder weitere Routinedatennutzungsprojekte mit anderen Zielrichtungen (z. B. Qualitätssicherung). Damit wurde eine Grundlage geschaffen, um über alle deutschen UK hinweg unmittelbar in der Krankenversorgung erhobene Routinedaten zu integrieren und für standortübergreifende Analysen zu nutzen.

Die Architektur basiert auf den in der MII [4] an allen deutschen UK etablierten DIZ sowie deren Steuerungs- und Datenaustauschprozessen. Eine semantische Harmonisierung der COVID-19-Daten wurde durch die Nutzung des GECCO-Datensatzes und dessen FHIR(Fast Healthcare Interoperability Resources)-Spezifikation erreicht [5]. Zur dezentralen Bereitstellung dieses Datensatzes wurde in jedem DIZ ein FHIR-Server etabliert. Für die Erhebung der Daten, die bisher noch nicht Bestandteil der elektronischen Krankenakte waren, wurden Datenerfassungssysteme (REDCap und DIS) mit standardisierten Data Dictionaries und einem vordefinierten Extraktionsprozess zur Generierung der FHIR-GECCO-Formate bereitgestellt. Für föderierte Machbarkeitsabfragen wurden diese FHIR-Server über zentral entwickelte Komponenten und sichere Datenverbindungen an ein Abfrageportal zur klinischen Charakterisierung von COVID-19-Patient*innen angebunden [6–8]. Auf Basis dieser dezentralen COVID-19-Forschungsdatenrepositorien konnten wissenschaftliche Erkenntnisse zum Verlauf der COVID-19-Pandemie und zu ihren Auswirkungen auf die stationäre Versorgung in den UK bereits im Sommer 2020 gewonnen werden [9–11].

Die Übermittlung der GECCO-Datensätze an die neu entwickelte zentrale Plattform erforderte die Erweiterung der Einwilligung des MII Broad Consent um ein NUM-RDP-spezifisches Modul. Die Einholung des Ethikvotums sowie die Implementierung dieser COVID-19-spezifischen Einwilligung an allen beteiligten UK erwies sich im Projektverlauf als eines der größten Hindernisse, weshalb die Strukturen und Prozesse zwischenzeitlich so weiterentwickelt wurden, dass zukünftig auch Datennutzungsvorhaben auf Basis eines Standard-MII-Broad-Consents unterstützt werden. Die Implementierung einer föderierten Treuhandstelle (federated Trusted Third Party [fTTP]; [12]) ermöglicht eine institutionenübergreifende sowie datenschutzkonforme Datensatzverknüpfung (PPRL) und stellt die Einhaltung der Anforderungen der Europäischen Datenschutzgrundverordnung (EU-DSGVO) sicher. Portale, über die Machbarkeitsanfragen gestellt werden, ermöglichen das Auffinden der verfügbaren Daten und sind u. a. generisch skalierbar konzipiert, sodass eine schnelle Erweiterbarkeit auf neue Datensatzstrukturen im Rahmen von Folgeprojekten der MII möglich war^9^ [13].

### Radiologische Plattform für Bildgebungsdaten (RACOON)

Die medizinische Bildverarbeitung im NUM wird im radiologischen multizentrischen Forschungsnetzwerk RACOON^10^ (Radiological Cooperative Network) adressiert. In dem 2020 ins Leben gerufenen Netzwerk sind alle radiologischen UK sowie die Technologiepartner Deutsches Krebsforschungszentrum Heidelberg (DKFZ), Fraunhofer MEVIS in Bremen, Technische Universität Darmstadt, Mint Medical GmbH und ImFusion GmbH zusammengeschlossen. War das Ziel von RACOON zunächst, die COVID-19-Pandemie besser zu verstehen und Behandlungsmöglichkeiten zu unterstützen, soll sich RACOON mittelbar zur Unterstützung von zahlreichen anderen medizinischen Anwendungsfällen entwickeln.

In RACOON werden medizinische Bilddatensätze wie Röntgenbilder und Computertomografien verarbeitet und geteilt. Um weiterreichende Erkenntnisse zu gewinnen, werden komplementäre Datensätze, bspw. Angaben zu Krankheitsverläufen in maschinenlesbarer Form, verarbeitet. Diese multimodalen Datensätze eröffnen ein breites Spektrum der Bilddatenanalyse und Methodenentwicklung.

In RACOON wird bspw. künstliche Intelligenz (KI) trainiert, um Assistenzfunktionen zu schaffen, die personalisierte Medizin und Präzisionsmedizin ermöglichen. Die Anwendungsfälle reichen damit von der detaillierten Befundung eines einzelnen Falls bis zur Kapazitätsplanung im Gesundheitswesen auf nationaler Ebene. RACOON ermöglicht insbesondere die Entwicklung und Bereitstellung homogener Datenerhebungs- und Analysemethoden. Die gewonnenen Erkenntnisse und Methoden können von Partnerstandorten schnell über das gesamte Projektnetzwerk verteilt werden.

**Figure 2:**
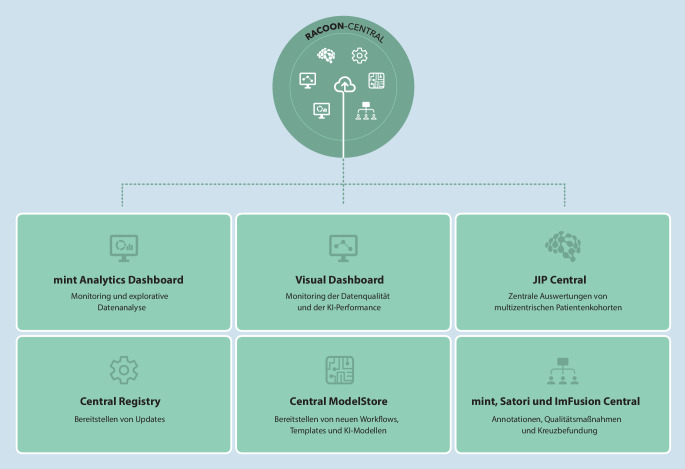

**Figure 3:**
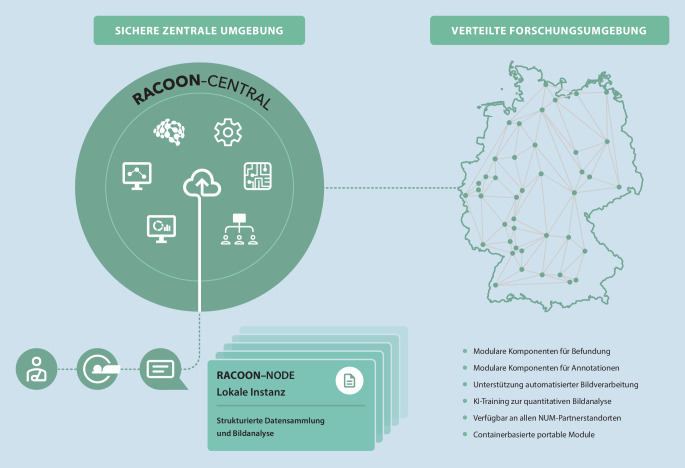

Für den bundesweiten Einsatz des Netzwerks bedarf es einer leistungsfähigen Infrastruktur und abgestimmter Definitionen zu gemeinsamen Informationsmodellen und Schnittstellen zwischen den Komponenten (Abb. 2). RACOON hat hierzu eine sichere, zentrale Umgebung und ein einzigartiges, hybrides Netzwerkinfrastrukturkonzept entwickelt, das verteilte Hardwareknoten (NODEs) an den beteiligten UK beinhaltet (Abb. 3).

Die RACOON-NODEs erlauben das Ausführen und Trainieren von KI-Modellen an allen Standorten. Alle Knoten sind gleichartig aufgebaut und ausgestattet, so können Ärzt*innen der Partnerinstitute multizentrische Forschungsprojekte über die hiermit ausgerollten Forschungsumgebungen initiieren bzw. an solchen teilnehmen.

Neben einer wirksamen und schnell adaptierbaren FIS ist eine gut strukturierte, repräsentative Datengrundlage die Voraussetzung für leistungsfähige KI-Entwicklung. Mittlerweile stehen in RACOON über 14.000 Fälle mit mehr als 8,8 Mio. Datenitems aus dieser initialen Datenerhebung im Netzwerk bereit. Hierzu wurden internationale und nationale Standards wie CO-RADS [14], COV-RADS [15], die Empfehlung der Arbeitsgruppe Thorax der Deutschen Röntgengesellschaft [16] und weitere aufgegriffen sowie auch neue radiologische Befundungsstandards abgestimmt. RACOON erlaubt hochqualitative strukturierte Auswertungen multimodaler Datensätze im multizentrischen Setting. Die RACOON-Datenmodelle enthalten Indizierungen auf gängige Systeme wie Radlex, SNOMED CT und LOINC [17, 18]. Die Interoperabilität innerhalb des NUM wird in RACOON mittels standardisierter Schnittstellen wie FHIR^11^ und DICOM^12^ umgesetzt. Die zugrundeliegende Architektur befähigt auch zu Echtzeitanalysen und Monitoring [19], sowohl dezentral für einzelne NODEs als auch im gesamten Netzwerk über die sichere zentrale Umgebung.

Die beispiellose Herausforderung der COVID-19-Pandemie und der kooperative Förderrahmen des NUM haben die Basis geschaffen, um die universitären Kräfte auf aktive Zusammenarbeit statt Kompetition auszurichten. Dabei stellen die unterschiedlichen datenschutzrechtlichen Voraussetzungen der einzelnen Bundesländer bundesweite Netzwerkprojekte vor erhebliche Herausforderungen, welche RACOON u. a. mit einem einheitlichen, rechtskonformen Datenschutzkonzept bewältigen möchte.

### ATKIN-Notaufnahmeregister (AKTIN@NUM)

Das AKTIN-Notaufnahmeregister wurde bereits vor der NUM-Gründung in einem BMBF-geförderten Verbundprojekt^13^ aufgebaut. Es handelt sich um eine interoperable, datenschutzkonforme Infrastruktur zur kontinuierlichen tagesaktuellen Nutzung von klinischen Routinedaten aus Notaufnahmen [20].

Wesentliches Ziel von AKTIN ist es, Versorgungsdaten aus der klinischen Routine verschiedener Krankenhäuser ohne Zusatzdokumentation für das medizinische Personal tagesaktuell verfügbar zu machen. Anwendungsszenarien für die Datenauswertung sind dabei die Versorgungsforschung, epidemiologische Fragestellungen, Qualitätssicherung und *Public Health Surveillance* [21–24]. Seit Juni 2020 werden die Wochenberichte der Notaufnahmesurveillance des RKI unter Nutzung der Daten aus dem AKTIN-Notaufnahmeregister veröffentlicht.^14^ Im Rahmen der NUM-Förderung wurden verschiedene wissenschaftliche Fragestellungen (Use Cases) mit unterschiedlichen Anforderungen an die Daten und an die Infrastruktur bearbeitet. Während z. B. für die *Public Health Surveillance* eine sehr schnelle Verfügbarkeit bei geringem Datenumfang notwendig ist, verlangen epidemiologische Fragestellungen eine größere Variablenanzahl mit höherem Detailgrad.

Um den Bedarfen der unterschiedlichen Anwendungsszenarien gerecht zu werden, wurden eHealth- bzw. Interoperabilitätsstandards umgesetzt. Der medizinische Dokumentationsstandard „Datensatz Notaufnahme“ der Deutschen Interdisziplinären Vereinigung für Intensiv- und Notfallmedizin (DIVI) e. V. wurde syntaktisch und semantisch standardisiert und bildet die Grundlage für eine interoperable sekundäre Nutzung der Routinedaten.

**Figure 4:**
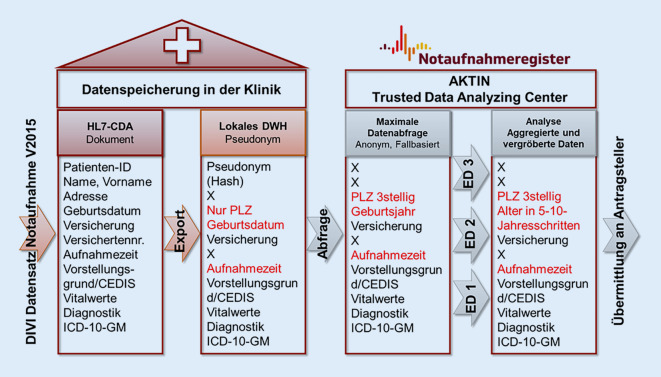

Eine dezentrale Registerinfrastruktur stellt die Einhaltung der EU-DSGVO-Richtlinien sicher. In den teilnehmenden Kliniken werden die Routinedaten zunächst in dem Klinischen Informationssystem der Notaufnahmen gespeichert. Über eine standardisierte, konsentierte IT-Schnittstelle (Health Level Seven Clinical Document Architecture – HL7 CDA) werden die Daten unter Nutzung von semantischen Interoperabilitätsstandards (ICD-10-GM [Internationale statistische Klassifikation der Krankheiten und verwandter Gesundheitsprobleme 10. Revision German Modification], OPS [Operationen- und Prozedurenschlüssel], LOINC [Logical Observation Identifiers Names and Codes] sowie proprietären Ersatzcodes als Substitution für die Terminologie SNOMED CT) in lokale Datawarehouses (DWHs) übertragen und pseudonymisiert gespeichert. Die lokalen DWHs kommunizieren regelmäßig mit dem zentralen AKTIN-Broker. Zweckgebunden können diese Daten über die zentrale Infrastruktur angefragt und an den Aggregator übermittelt werden [25, 26]. Der Datenschutz wird durch technisch-organisatorische Maßnahmen und einen sukzessive steigenden Anonymisierungsgrad der Daten sichergestellt (Abb. 4). Forscher*innen aller öffentlichen Forschungsinstitutionen und der teilnehmenden Kliniken sind berechtigt, Anträge auf Datenauswertung zu stellen.

Insbesondere die dynamischen Entwicklungen in der COVID-19-Pandemie haben gezeigt, wie groß der Mehrwert einer schnellen und zeitlich begrenzten Anpassung der Routinedokumentation in den Notaufnahmen ist. Es wird angestrebt, bereits entwickelte theoretische Konzepte zur Erhöhung der Agilität und Flexibilität des Datensatzes und der Datenerhebung weiter umzusetzen.

Die Erfahrungen aus dem Aufbau und der Verbreitung des AKTIN-Notaufnahmeregisters zeigen, dass die Implementierung einer Infrastruktur unter Nutzung inhaltlicher und technischer Standards herausfordernd ist. Inhaltliche und technische Standards in der Fläche durchzusetzen bedarf aufgrund unterschiedlicher regulatorischer Anforderungen, der Vielfalt von IT-Systemen für und in den Notaufnahmen und fehlender gesetzlicher Vorgaben umfassender zeitlicher und finanzieller Ressourcen. Ein wesentlicher Vorteil ist die Architektur des AKTIN-Notaufnahmeregisters, mit welcher eine systemunabhängige Integration möglich ist. Dies ist die Voraussetzung für eine weitreichende bzw. bundesweit flächendeckende Skalierung.

### Plattform für genomische Virussurveillance (GenSurv)

GenSurv ist eine zentrale Plattform zur Speicherung, Analyse und Visualisierung von SARS-CoV-2-Sequenzierungsdaten. Das Ziel ist die Unterstützung der bundesweiten Sequenzierungsaktivitäten der UK, des RKI und weiterer sequenzierender Einrichtungen sowie die Nutzbarmachung dieser Informationen für alle Beteiligten über eine entsprechende Infrastruktur. Neben der Erfassung und Speicherung der genomischen Forschungsdaten in dieser zentralen technischen Infrastruktur, dem *Data Hub,* soll die Plattform auf verschiedene Zielsetzungen ausgerichtete, bestgeeignete Beprobungsstrategien und phänotypische Charakterisierungen erarbeiten. Aufgrund ihrer besonderen Bedeutung für die öffentliche Gesundheit und Patient*innenversorgung [27–30] soll die Infrastruktur kontinuierlich ausgebaut werden und ab 2023 weitere relevante Erreger aufnehmen.

Erfasst werden Rohdaten und assemblierte virale Genomsequenzen (komplementär zum elektronischen Sequenzdatendaten-Hub [DESH] des RKI^15^) als Voraussetzung für (i) die frühzeitige Erkennung der Evolution von verschiedenen Virusvarianten mit Potenzial für Immun- oder Impfresistenz oder geänderter biologischen Eigenschaften, (ii) die Charakterisierung von intra- und interindividueller genetischer Variabilität sowie für (iii) die Algorithmenentwicklung und Qualitätskontrolle bei der Genomassemblierung (Pipeline) [31]. Eine solche optimierte Assemblierungspipeline wird im Data Hub zur Verfügung gestellt und auf übertragene Datensätze zur Qualitätskontrolle und periodischen Qualitätsevaluation angewendet.

Die Infrastruktur kann vom gesamten NUM-Netzwerk zur Speicherung und Analyse von SARS-CoV-2-Sequenzierungsdaten genutzt werden. Die Komponenten des Data Hubs stehen überdies der wissenschaftlichen Gemeinschaft und weiteren Stakeholdern zur Verfügung, inkl. des Öffentlichen Gesundheitsdienstes und kommerzieller diagnostischer Labore. Ein User-Support wird unter Einsatz eines „Issue-Tracking-Systems“^16^ abgewickelt. Die Datenspeicherung im Data Hub findet innerhalb der „German Network for Bioinformatics Infrastructure“ (de.NBI)^17^ statt und basiert damit implizit auf den für de.NBI entwickelten Konzept zur sicheren und dauerhaften Datenspeicherung.

**Figure 5:**
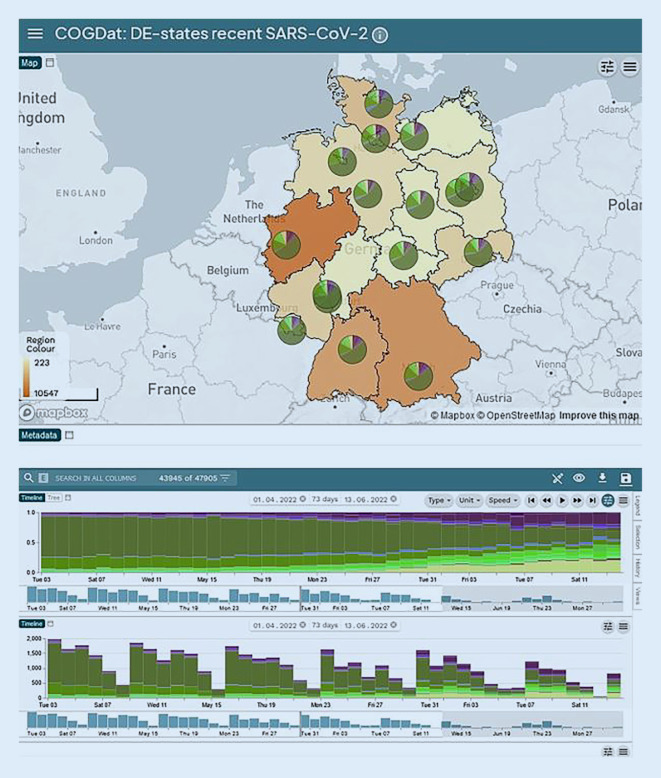

Beim Data Hub handelt es sich um CoGDat (SARS-CoV‑2 Genomics Data Platform), eine gemeinsame Initiative von Forschenden, die sich für die Verfügbarmachung von Sequenzdaten im Rahmen des NUM vernetzt haben. Für die Datenerfassung verwendet CoGDat das eigens dafür entwickelte Open-Source-Datenportal DataMeta, welches das Hochladen von Daten über ein Web-Interface im Browser sowie automatisiertes Hochladen von Daten durch eine Representational State Transfer (REST)-Schnittstelle (API) erlaubt. Das Hochladen von Sequenzierungsdaten (FASTA- und FASTQ-Formate) ist aus Datenschutzgründen derzeit noch nicht möglich. CoGDat verfügt über automatisierte Datenverarbeitungspipelines zur Analyse von Pathogen-Sequenzierungsdaten (u. a. aus der nf-core-Initiative) und eine auf dem Microreact-Framework basierende Visualisierungsplattform zur Darstellung und Interpretation von phylogenetischen Daten (Abb. 5). Diese Tools werden in unmittelbarer Zukunft öffentlich verfügbar gemacht.^18^

Ein Zwischenfazit ist, dass die Schaffung von klaren rechtlichen Rahmenbedingungen förderlich für die Verarbeitung von Pathogen-Sequenzierungsdaten wäre. Zentral wäre auch die Einbindung der technischen Infrastruktur in personelle und prozessuale Infrastrukturen. Deren Zielsetzung ist die Entwicklung eines Konzeptes zu bestgeeigneten Samplingstrategien und die Priorisierung der Aufnahme weiterer Infektionserreger gemäß ihrer Relevanz und des Mehrwertes genomischer Surveillance. Die Ausarbeitung erfolgt in Zusammenarbeit mit Vertreter*innen des RKI. Mitte 2022 fand dazu der erste Workshop statt, zu dem auch ein Expert*innenboard einberufen wurde.

## Weiterentwicklung der Forschungsinfrastrukturen im Netzwerk Universitätsmedizin

Das NUM hat aus dem Stand komplexe FIS aufgebaut. Damit konnten auch im internationalen Vergleich beachtliche Erfolge in der Gewinnung von Forschungsdaten zu COVID-19 erzielt werden. Z. B. hat NUKLEUS mit knapp 6.000 rekrutierten Patient*innen den Aufbau einer der global größten tief phänotypisierten Kohorten ermöglicht. RACOON ist eine weltweit einzigartige Plattform zum standortübergreifenden Teilen mit bereits über 14.000 hochstrukturierten Daten aus der Bildgebung. Angesichts des Zeitdrucks und der schwierigen Ausgangslage sind dies große Erfolge. Dennoch ist der Aufbau der FIS weiterhin „work in progress“. In fast allen Bereichen bestehen noch Weiterentwicklungsmöglichkeiten. Dies betrifft so unterschiedliche Themen wie die Rekrutierungsgeschwindigkeit bei Studienpatient*innen, die Einbindung von Patient*innenvertretungen in die Governance, die Zusammenführung von Daten („record linkage“) aus verschiedenen FIS, die Einbindung von Omics-Daten, die konsequente Implementierung von Standards oder die Skalierung der Plattformen hinsichtlich der Anbindung möglichst vieler Standorte.

Diese Vorhaben sind fachlich, technisch und organisatorisch komplex. Sie bedürfen einer intensiven wissenschaftlichen Begleitung, denn in aller Regel existiert für diese Herausforderungen auch im internationalen Vergleich keine Best Practice, die ohne großen Aufwand ausgerollt werden könnte. Zudem müssen sich die FIS durch die im Gesundheitssystem voranschreitende Digitalisierung und den medizinisch-technischen Fortschritt dynamisch immer wieder an neue technische Entwicklungen und Datenquellen anpassen. Das NUM steht somit für die kommenden Jahre vor der doppelten Herausforderung, seine FIS zu konsolidieren und gleichzeitig weiterzuentwickeln. Hierfür bedarf es einerseits der notwendigen wissenschaftlichen Freiheitsgrade für die Entwickler*innen, Datenerzeuger*innen und Datennutzer*innen. Andererseits bedarf es einer zentralen Koordination und einheitlichen Rahmensetzung für diese Aktivitäten.

**Figure 6:**
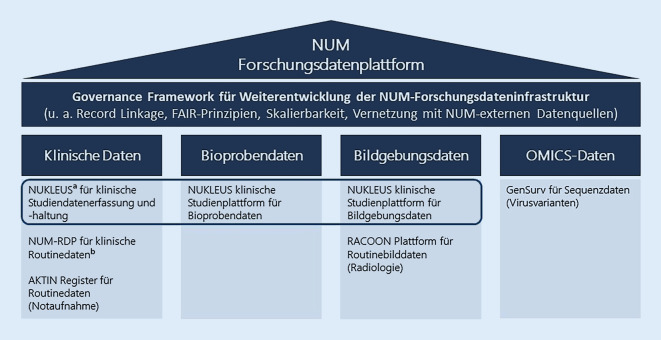

Abb. 6 zeigt den aktuellen Stand der FIS und deren geplante kontinuierliche und aufeinander abgestimmte Weiterentwicklung. Die Säulen repräsentieren einzelne Datenarten, denen die FIS jeweils zugeordnet sind. Ziel ist es, diese Säulen mittels einer Dachstruktur übergreifend zu verbinden. Die Governance für eine solche Dachstruktur befindet sich aktuell im Aufbau. Alle im NUM schon existierenden und weitere noch hinzukommende Infrastrukturprojekte sollen im Rahmen einer solchen Dachstruktur synergistisch und komplementär mit möglichst weitgehend harmonisierten technischen Grundlagen weiterentwickelt werden.

Die Vernetzung mit weiteren, NUM-externen Datenquellen gehört ebenfalls zum Aufgabenspektrum einer solchen Dachstruktur. Ansonsten bestünde das Risiko, dass neue, untereinander nicht verbundenen Datensilos entstehen, denn medizinische Forschungsdaten sind ebenso wie die Gemeinschaft der Forschenden extrem heterogen und gehen weit über „Real World Data“ (Daten aus dem Versorgungsalltag) hinaus. Dies wird aus der Beschreibung der fünf FIS deutlich, die jeweils unterschiedliche Datenarten, Wege der Datengewinnung (prospektiv vs. retrospektiv) sowie Datenentstehungssettings (Notaufnahmen, Radiologien etc.) beinhalten.

Die synergistische Verknüpfung unterschiedlicher FIS im NUM wird im Jahr 2023 durch Hinzufügen zweier weiterer FIS verstärkt. Dabei handelt es sich um NATON (Nationales Obduktionsnetzwerk) und die DIZ der MII.

Dank ihrer technologischen Offenheit bieten die FIS eine hervorragende Ausgangsbasis, um die Gewinnung, Haltung und Bereitstellung biomedizinischer Forschungsdaten auch über die Universitätsmedizin hinaus zu organisieren und damit Breite und Repräsentativität der Datengrundlagen zu verbessern. Erste nichtuniversitäre Krankenhäuser und Vertragsarztpraxen sind in einzelnen NUM-Projekten bereits angebunden. Die großflächige Anbindung nichtuniversitärer Leistungserbringer ist jedoch technisch anspruchsvoll und insbesondere für die anzubindenden Partner mit erheblichen Aufwänden verbunden, die vielerorts nicht ohne Weiteres leistbar sind. Hier gilt es, in den nächsten Jahren niedrigschwellige Lösungen zu entwickeln, die nichtuniversitären Partnern eine möglichst aufwandsarme Anbindung erlauben. Perspektivisch angestrebt ist ebenfalls die Integration von Daten aus dem Bereich „Consumer Health“ (z. B. aus Wearables) sowie von Daten aus Gesundheitsämtern, Registern oder dem Forschungsdatenzentrum des Bundesinstituts für Arzneimittel und Medizinprodukte (BfArM).

## Fazit

Zusammenfassend ist festzuhalten, dass im Netzwerk Universitätsmedizin (NUM) in sehr kurzer Zeit Forschungsinfrastrukturen (FIS) aufgebaut werden konnten, welche die Forschung bereits heute mit qualitativ hochwertigen Daten unterstützen. Dennoch besteht enormes Weiterentwicklungspotenzial und auch -bedarf. Das NUM bietet bzgl. Finanzierung, Governance und Organisationsstruktur den notwendigen Rahmen, um in Deutschland in den nächsten Jahren eine international sichtbare, hochqualitative FIS aufzubauen, die im Sinne des Open-Science-Gedanken von der wissenschaftlichen Community breit genutzt werden kann.

## Footnotes

1 https://www.bmbf.de/bmbf/shareddocs/pressemitteilungen/de/karliczek-netzwerk-universitae-e-forschungsarbeit-zu-covid-19.html (aufgerufen am 11.10.2022).

2 z. B. Erkrankungsverlauf, Schutzkonzepte, Surveillance-Konzepte, Impfung und Immunität.

3 https://www.netzwerk-universitaetsmedizin.de/projekte (aufgerufen am 02.08.2022).

4 FAIR Data Principles – die Daten müssen leicht auffindbar (findable), zugänglich (accessible), interoperabel (interoperable) und wiederverwendbar (reusable) sein.

5 https://www.netzwerk-universitaetsmedizin.de/gecco-data-set (aufgerufen am 02.08.2022).

6 zentrale Datenhaltung von GECCO-Daten; Betrieb und Weiterentwicklung der in CODEX (Teilprojekt der 1. Förderperiode) etablierten Strukturen.

7 Erweiterung von CODEX um die Möglichkeit, projektbezogene Verbundforschung auf Basis des MII Broad Consent zu realisieren.

8 auf Basis des MII Broad Consent.

9 Die einzelnen Komponenten der NUM-RDP wurden als Open-Source-Software bereitgestellt.

10 https://racoon.network (aufgerufen am 02.08.2022).

11 License – FHIR v4.3.0: http://hl7.org/fhir/license.html (aufgerufen am 25.07.2022).

12 DICOM https://www.dicomstandard.org (aufgerufen am 25.07.2022).

13 Verbundprojekt „Verbesserung der Versorgungsforschung in der Akutmedizin in Deutschland durch den Aufbau eines nationalen Notaufnahmeregisters“, hervorgegangen aus dem Aktionsbündnis für Informations- und Kommunikationstechnologie in Intensiv- und Notfallmedizin, 01KX18017: Laufzeit von 2013 bis 2019.

14 https://www.rki.de/DE/Content/Institut/OrgEinheiten/Abt3/FG32/sumo/sumo.html (aufgerufen am 03.08.2022).

15 https://www.rki.de/DE/Content/InfAZ/N/Neuartiges_Coronavirus/DESH/DESH.html (aufgerufen am 02.08.2022).

16 Hierbei handelt es sich um eine Software, die die eingehenden Daten sammelt, clustert und an die entsprechende Funktionsstelle weiterleitet.

17 de.NBI bietet Lösungen wie z. B. bioinformatische Werkzeuge für die Auswertung großer Datenmengen mittels komplexer Analysemethoden.

18 www.cogdat.de (aufgerufen am 02.08.2022).
